# Reply to Gobert et al.: The need for more research to understand changes in new pelagic sargassum ecosystem during its advection

**DOI:** 10.1073/pnas.2412718121

**Published:** 2024-08-08

**Authors:** Carla Botelho Machado, Robert Marsh, Jessica K. Hargreaves, Hazel A. Oxenford, Gina-Marie Maddix, Dale F. Webber, Mona Webber, Thierry Tonon

**Affiliations:** ^a^Centre for Novel Agricultural Products, Department of Biology, University of York, York YO10 5DD, United Kingdom; ^b^School of Ocean and Earth Science, University of Southampton, Southampton SO14 3ZH, United Kingdom; ^c^Department of Mathematics, University of York, York YO10 5DD, United Kingdom; ^d^Centre for Resource Management and Environmental Studies, University of the West Indies, Cave Hill BB 11000, Barbados; ^e^Centre for Marine Sciences, Department of Life Sciences, University of the West Indies, Mona Kingston 7, Jamaica; ^f^Department of Life Sciences, University of the West Indies, Mona Kingston 7, Jamaica

Gobert et al. (1) present new data on three elements, Fe, Al, and Mn, in sargassum harvested in June 2021 near the French Caribbean Islands Guadeloupe and Martinique (FCIs). They also analyzed the content of these elements in sargassum incubated for 21 d in the coastal area of Baie du Robert (Martinique). Using this information, they challenged our suggestion that exposure of sargassum to ash after La Soufrière volcanic eruption (April 2021) may have contributed to increasing the content of these three metals in biomass harvested in Jamaica from August 2021 (2). Previously, Gobert et al. (3) suggested that coastal waters received terrigenous inputs, including Al, Fe, and Mn, increasing the content of these elements in sargassum harvested in coastal areas. Considering the high Fe, Al, and Mn content in La Soufrière volcanic ash, together with the potential exposure of sargassum to ash for 50 d, it made sense to us to suggest that this unusual condition could alter the elemental composition of sargassum biomass arriving in late summer in Jamaica (2).

Compared to Jamaica, FCIs are much closer to the sargassum potentially affected by volcanic ash, so sargassum arriving offshore of these islands will have had less time (2 mo, by June) to assimilate elements than the sargassum reaching Jamaica (after 4 mo). Additionally, there is still uncertainty in how long, and along which pathways, the sargassum was sharing ash-enriched surface water (2). A further caveat with the June samples is that the potentially ash-affected sargassum arrived more quickly in FCIs than Gobert et al. (1) supposed, probably in May. Downstream at Jamaica, months later, dispersal along the transport pathway and during extended time may have more clearly impacted sargassum elemental composition. Moreover, much of the sargassum arriving in FCIs, the northern sector of the Lesser Antilles, is likely to come from outside the area downwind of La Soufrière (4), so a sampled mix of sargassum may be largely unaffected by ash deposition. Particle tracking data presented in Machado et al. (2) suggested that in 2021 a high proportion of sargassum reached Jamaica via the western boundary current after potential exposure to volcanic ash. Finally, considering the trajectory of sargassum reaching Jamaica, it is unlikely this biomass spent up to 21 d confined and growing in a coastal zone environment similar to the Baie du Robert (1). At the Jamaican collection site, sargassum beaches “directly” on each tide after short residence time in coastal waters, with therefore little opportunity to accumulate terrigenous metals.

To conclude, we suggest that more coordinated and interdisciplinary research is needed to investigate the biology and the ecology of pelagic sargassum during its advection. This could involve GPS tracking of algae rafts during a full season of sargassum to inform regular sampling and then investigation of morphotype abundance, fauna, and microbiota associated with the rafts and of (bio)chemical composition of the algae. Integrating such information will advance the understanding and prediction of new pelagic sargassum ecosystems under changing environmental conditions.
